# Omenn Syndrome in Two Infants with Different Hypomorphic Variants in Janus Kinase 3

**DOI:** 10.1007/s10875-024-01699-5

**Published:** 2024-04-10

**Authors:** Christo Tsilifis, Jarmila Stremenova Spegarova, Ross Good, Helen Griffin, Karin R. Engelhardt, Sophie Graham, Stephen Hughes, Peter D. Arkwright, Sophie Hambleton, Andrew R. Gennery

**Affiliations:** 1 Paediatric Haematopoietic Stem Cell Transplant Unit, Great North Children’s Hospital, Victoria Wing, Royal Victoria Infirmary, Newcastle Upon Tyne, NE1 4LP UK; 2 Translational and Clinical Research Institute, Newcastle University, Newcastle Upon Tyne, UK; 3 Faculty of Medical Sciences, Medical School, Newcastle University, Newcastle Upon Tyne, UK; 4 Lydia Becker Institute of Immunology and Inflammation, University of Manchester, Manchester, UK

**Keywords:** Severe combined immunodeficiency, Omenn syndrome, Haematopoietic stem cell transplant, Janus kinase 3

## Abstract

Biallelic null or hypomorphic variants in *JAK3* cause SCID and less frequently Omenn syndrome. We investigated homozygous hypomorphic JAK3 mutations in two patients, and expression and function of a novel JAK3^R431P^ variant in Omenn syndrome. Immunophenotyping of PBMC from the patient with the novel JAK3^R431P^ variant was undertaken, by flow cytometry and Phosflow after stimulation with IL-2, IL-7, and IL-15. JAK3 expression was investigated by Western blotting. We report two patients with homozygous hypomorphic *JAK3* variants and clinical features of Omenn syndrome. One patient had a previously described JAK3^R775H^ variant, and the second had a novel JAK3^R431P^ variant. One patient with a novel JAK3^R431P^ variant had normal expression of JAK3 in immortalised EBV-LCL cells but reduced phosphorylation of STAT5 after stimulation with IL-2, IL-7, and IL-15 consistent with impaired kinase activity. These results suggest the JAK3^R431P^ variant to be hypomorphic. Both patients are alive and well after allogeneic haematopoietic stem cell transplantation. They have full donor chimerism, restitution of thymopoiesis and development of appropriate antibody responses following vaccination. We expand the phenotype of hypomorphic JAK3 deficiency and demonstrate the importance of functional testing of novel variants in disease-causing genes.

**Supplementary Information:**

The online version contains supplementary material available at 10.1007/s10875-024-01699-5.

## Introduction

Severe combined immunodeficiency (SCID) usually results from recessive loss of function variants in genes involved in T-lymphocyte development, and is characterised immunologically by absent or low and dysfunctional T-cells, and variably affected B- or NK-cell number and function, depending on the pathway affected [1, 2]. Patients with typical SCID present in the first year of life with failure to thrive, diarrhea, and recurrent infections, including opportunistic infections such as *Pneumocystis jirovecii* pneumonia [2]. Absence of T-cells may also allow the transplacental engraftment of maternally-derived T-cells, which are typically oligoclonal and alloreactive and manifest as graft-versus-host disease (GvHD) with lichenified erythroderma, alopecia, diarrhea, and lymphadenopathy [3]. These clinical features are shared with Omenn syndrome (OS), which results from an incomplete block of T-cell development. Low numbers of autologous T-cells emerge from the thymus and show oligoclonal expansion with end-organ infiltration and damage. Since Omenn’s first description in an Irish family in 1965 [4], the syndrome has expanded to include T-lymphocytosis with an activated phenotype and oligoclonal expansion of TCRVβ families, raised serum IgE, peripheral eosinophilia, and absent or reduced T-cell proliferative response to antigens [5]. OS is predominantly associated with hypomorphic mutations in recombination-activating genes 1 and 2 (*RAG1/2*) [6], but has also been described in patients with mutations in *DCLRE1C* [7] and, less commonly, *IL7RA* [8]*.* All of these genes play critical roles during thymocyte development but are more or less redundant for mature T-cell effector function. Other genes implicated in SCID include those affecting expression of proteins involved in transducing TCR and cytokine signals, such as *IL2RG* and *JAK3.* Omenn syndrome as a manifestation of JAK3 SCID has only been reported in one previous patient [9].

Null or hypomorphic variants in *JAK3* are associated with SCID of T-B + NK- phenotype [10], similar to X-linked SCID caused by loss of function of *IL2RG*, encoding the common gamma chain of type I cytokine receptors [11]. As a member of the Janus kinase family of proteins, JAK3 transduces signals from interleukins IL-2, IL-4, IL-7, IL-9, IL-15, and IL-21, critical for regulating lymphocyte expansion and differentiation [12–14].

We report the presentation and clinical course of two unrelated patients with OS, each of whom bore different homozygous mutations in *JAK3*. We demonstrate that the novel JAK3^R431P^ variant protein is normally expressed, however its signalling function in response to interleukin stimulation is diminished. Our findings emphasise the genetic heterogeneity of OS, the phenotypic spectrum associated with hypomorphic JAK3 deficiency and the importance of functional analysis to clarify the significance of novel missense variants in known disease genes.

## Methods

Clinical data were collected retrospectively from medical records. Both patients’ families gave written consent for approved research including data collection, analysis, and publication. (REC reference 20/NE/0044).

### Sample Collection and Generation of Patient’s Primary EBV-LCL Cell Line

Peripheral blood mononuclear cells (PBMC) were isolated from EDTA blood samples using Lymphoprep (StemCell Technologies, 07851) density gradient centrifugation as per manufacturer’s instructions.

EBV-LCL cell lines were generated by immortalization of isolated PBMC by infecting the cells with EBV supernatant produced by the B95-8 marmoset cell, and cyclosporine A (1ug/ml). Cells were subcultured in RPMI-1640 culture medium (Sigma Aldrich, R0883) supplemented with 10% (v/v) foetal calf serum (FCS, Gibco, 10270–106), 1% (v/v) Penicillin/Streptomycin (100 U/mL and 100 μg/mL respectively; Sigma Aldrich, P0781) and 1% (v/v) L-Glutamine (2 mM; Sigma Aldrich, G7513), referred as RPMI10.

### Immunophenotyping by Flow Cytometry

PBMC were thawed at 37°C, transferred into pre-warmed complete RPMI10 culture medium, pelleted by centrifugation, resuspended in RPMI10, and left to rest at 37˚C for 1.5 h. Cells were stained with a cocktail of cell surface antibodies in FACS buffer (PBS + 2% FCS) for 30min at room temperature (RT) in the dark. Cells were washed and resuspended in FACS buffer, 7AAD viability dye (Biolegend) was added, and cells were acquired on a BD FACSymphony A5 flow cytometer (BD Biosciences). Data were analysed by FlowJo V10 (BD Biosciences). The following anti-human flow cytometry antibodies were used: TCR-gd-FITC (B1, Biolegend), CD19-PerCP-Cy5.5 (HIB19, Biolegend), CD14-PE (M5E2, Biolegend), CD56-PE-CF594 (NCAM16.2, BD Biosciences), CCR7-PE-Cy7 (G043H7, Biolegend), PD-1-AF700 (EH12.2H7, Biolegend), CD45RO-APC-Cy7 (UCHL1, BD Biosciences), CD127-BV421 (A019D5, Biolegend), CD28-BV480 (CD28.2, BD Biosciences), CD20-BV605 (L27, BD Biosciences), CD16-BV650 (3G8, BD Biosciences), HLA-DR-BV711 (L243, Biolegend), CD4-BV786 (SK3, Biolegend), CD3-BUV395 (UCHT1, BD Biosciences), CD8-BUV496 (RPA-T8, BD Biosciences), CD25-BUV737 (2A3, BD Biosciences).

### Phosphoflow Cytometry

PBMC and EBV-LCL cells were rested in serum free RPMI culture media for 2hr or overnight in incubator at 37°C, respectively. Cells were stained with fixable viability dye Zombie yellow (Biolegend), CD16-BV421 (3G8, BD Biosciences), CD20-BV510 (2H7, Biolegend) and CD8-BUV496 (RPA-T8, BD Biosciences) for 30 minutes. Following the pre-staining, cells were divided into the appropriate number of FACS tubes and stimulated separately with IL-2, IL-7, IL-15 (all 100ng/ml, ThermoFisher Scientific), and IL-21 (50ng/ml, Miltenyi Biotec) for 15min at 37˚C. Cells were fixed 1:1 using Cytofix buffer (BD Biosciences) for 20min at RT. Permeabilization was achieved by adding ice-cold PermIII buffer (BD Biosciences), and incubation on ice for 20 min. After repeated washing steps with FACS buffer, cells were stained with antibodies: pSTAT1-AF488 (pY701, 4a, BD Biosciences), CD19-PerCP-Cy5.5 (HIB19, Biolegend), pSTAT3-PE (pY705, 4/P-STAT3, BD Biosciences), FOXP3-PE-Dazzle594 (206D, Biolegend), pSTAT5-AF647 (pY694, 47/Stat5(pY694), BD Biosciences), CD56-BV711 (5.1H11, Biolegend), CD4-BV786 (SK3, Biolegend), CD3-BUV395 (UCHT1, BD Biosciences) and CD25-BUV737 (2A3, BD Biosciences) for 60 min at RT in the dark. The staining panel for EBV-LCL cells was reduced to pSTAT antibodies, Zombie Yellow, and B cell markers CD19 and CD20. Samples were acquired on a BD FACSymphony A5 flow cytometer (BD Biosciences) and analyzed using FlowJo software. For data analysis, mean fluorescence intensity of unstimulated cells was subtracted from values after interleukin stimulation.

### JAK3 Protein Expression by Western Blotting

EBV-LCL cells were washed in PBS and lysed in lysis buffer for 15min [50 mM Tris–HCl (pH 7.5), 150 mM NaCl, 1% Nonidet P-40, 0.1% SDS, 0.5% Na-Deoxycholate] containing 100 mM dithiothreitol (Merck), 1 × complete protease inhibitor cocktail (Roche), 1 × PhosSTOP phosphatase inhibitors (Roche), and 1 × NuPAGE Loading Buffer (Thermo Fisher Scientific). Lysates were denatured at 95°C for 10 min before being subjected to 4–12% tris–glycine polyacrylamide gel (Thermo Fisher Scientific) electrophoresis in 1 × SDS NuPAGE MOPS Running Buffer (Thermo Fisher Scientific). Proteins were transferred to 0.45-mm Immobilon®-P polyvinyl difluoride membranes (Thermo Fisher Scientific) in 1 × NuPAGE Tris–Glycine Transfer Buffer supplemented with 20% Methanol. Membranes were blocked for 60 min in 5% bovine serum albumin in tris-buffered saline with 0.1% Tween (TBS-T) buffer before overnight immunostaining at 4°C with mouse anti-human JAK3 (Cell Signaling, 5481S, 1:1000) and rabbit anti-human GAPDH antibodies (Cell Signaling, 8884S, 1:5000). Membranes were washed in TBS-T and stained with secondary antibodies anti-mouse IgG HRP-linked (Cell Signaling, 7076S, 1:3000) and anti-rabbit IgG HRP-linked (Cell Signaling, 7074S, 1:5000) for 1h at RT. Membranes were developed with Immobilon ECL Ultra Western Substrate solution (Merck), imaged on a LI-COR Odyssey Fc (LI-COR) and Image Studio software was used for analysis.

### Statistical Analysis

For experiments with multiple repeats, data are expressed as the mean ± standard deviation. Where appropriate, statistical comparisons between Controls and Patient P2 data were calculated using unpaired t-test carried out through GraphPad Prism software with probability values (P) of P<0.05 designated significant.

## Results

### Omenn Syndrome-Like Patients with JAK3 Mutation

Patient 1 (P1), a male infant, was born at 40 weeks gestation following a normal pregnancy to consanguineous parents. There was no family history of immunodeficiency. Aged 8 weeks, he developed oral candidiasis, chronic diarrhoea, and recurrent chest infections, leading to faltering growth. He received immunisations as per schedule including Bacille Calmette-Guérin (BCG) vaccination. He subsequently developed alopecia with an erythematous, lichenified rash and axillary lymphadenopathy. Initial immunological investigations revealed a total lymphocytosis (11,060 cells/microlitre) with low CD3 + CD8 + T-cells (296 cells/microlitre) and absent naïve T-cells but preserved NK cells numbers (Table 1). There was no maternofoetal engraftment on cytogenetic testing. Flow phenotyping for TCRVβ usage showed expansion of certain subsets and absence of others, consistent with oligoclonality, and his lymphocytes had impaired proliferation following incubation with phytohemagglutinin. Immunoglobulin concentrations were low (IgA: 0.05g/L, IgM: 0.27g/L, IgG < 0.3g/L) except IgE, which was elevated (640kU/L). Virological testing showed rhinovirus and parainfluenza 4 in nasopharyngeal secretions, and sapovirus in his faeces. Due to the dermatological features of OS, he was commenced on topical tacrolimus and clobetasone 0.05%, and oral rifampicin, isoniazid and ethambutol were given for BCGosis.

**Table 1:** Patient cellular and humoral immunology at clinical presentation and at latest follow-up

	P1	P2	Normal
Presentation			
CD3 + (cells/microlitre)	11060	6681	(2300 – 7000)
CD3 + CD4 + (cells/microlitre)	10053	6668	(1400 – 5300)
CD3 + CD4 + CD45RA + (cells/microlitre)	Absent	Absent	
CD3 + CD8 + (cells/microlitre)	296	69	(400 – 2200)
CD3 + CD4 + CD45RA + (cells/microlitre)	Absent	Absent	
CD4:CD8 ratio	33.9	96.6	(0.9 – 3.9)
CD19 + (cells/microlitre)	2948	2074	(600 – 3000)
CD16 + 56 + (cells/microlitre)	479	238	(100 – 1400)
TCR-Vβ families	Oligoclonal	Oligoclonal	
IgA (g/L)	< 0.05	0.16	(0.1 – 0.5)
IgG (g/L)	< 0.3	2.0	(2.4 – 8.8)
IgM (g/L)	0.27	0.41	(0.2 – 1.0)
IgE (IU/ml)	640	> 50000	(0 – 15)
Latest follow-up			
Donor chimerism	CD3 + 95% CD15 + 9% CD19 + 27%	CD3 + 90% CD15 + 25% CD19 + 32%	
CD3 + (cells/microlitre)	2596	4860	(2300 – 7000)
CD3 + CD4 + (cells/microlitre)	1100	2691	(1400 – 5300)
CD3 + CD4 + CD45RA + (cells/microlitre)	493	729	
CD3 + CD8 + (cells/microlitre)	1045	1980	(400 – 2200)
CD3 + CD4 + CD45RA + (cells/microlitre)	519	437	
CD4:CD8 ratio	1.0	1.36	(0.9 – 3.9)
CD19 + (cells/microlitre)	633	1470	(600 – 3000)
CD16 + 56 + (cells/microlitre)	343	152	(100 – 1400)
IgA (g/L)	0.77	0.28	(0.1 – 0.5)
IgG (g/L)	9.3	6.1	(2.4 – 8.8)
IgM (g/L)	0.87	1.55	(0.2 – 1.0)

At 6 months of age, P1 underwent a bone marrow transplant from an HLA-identical sibling, following conditioning with treosulfan (36g/m^2^), fludarabine (150mg/m^2^) and serotherapy with alemtuzumab (1mg/kg). He received ciclosporin A and mycophenolate mofetil as GvHD prophylaxis. Peri-transplant, his course was complicated by engraftment pneumonitis which required oxygen, methylprednisolone, and a dose of infliximab prior to resolution, along with immune reconstitution against BCG presenting as axillary lymphadenitis requiring antimycobacterial therapy and incision and drainage. He received pre-emptive treatment with ganciclovir between days + 39 and + 70 post-HSCT for cytomegalovirus (CMV) viraemia. Two years post-transplant, whole exome sequencing of the patient’s germline (fibroblast) DNA revealed a homozygous pathogenic variant (c.2324G > A, p.R775H) in *JAK3*. This variant results in substitution of arginine for histidine at position 775 in the JH2 pseudokinase domain of the JAK3 protein, and has been reported in two other patients [9, 15]. No other pathogenic variants were identified in known IEI genes. Now 10 years post-transplant, P1 is well, off immunoglobulin and infection-free with normal lymphocyte subsets and stable mixed donor chimerism (CD3 + : 95%, CD19 + : 27%, CD15 + : 9%). At latest clinical review, P1’s alopecia has resolved and he has normal skin with no features of OS, and has had antimicrobial prophylaxis discontinued.

Patient 2 (P2), a female infant, was born at 40 weeks gestation as the first child to consanguineous parents of Syrian origin, with no family history of immunodeficiency. She did not receive BCG vaccination at birth. She presented aged 3 weeks with a persistent maculopapular rash which developed into erythroderma unresponsive to emollients, and initial investigations identified eosinophilia and total lymphocytosis (9,037 cells/microlitre) despite low CD3 + CD8 + T-cells (69 cells/microlitre) and absent naïve T-cells (Table 1). NK and B cell numbers were within normal limits. T-cell receptor (TCR) staining identified expansion of the TCRVβ-5.1, -14, and -20 subsets with several absent Vβ subsets. Maternofoetal engraftment studies were negative. Prior to HSCT, she required treatment with ganciclovir for CMV and human herpesvirus-6 (HHV6) viraemia. She had persistent fever despite broad-spectrum antibiotics and antifungal treatment, which resolved following intravenous methylprednisolone and serotherapy with anti-thymocyte globulin. Aged 5 months, P2 underwent HSCT using her 12/12 HLA-matched maternal grandfather as donor. She received a peripheral blood stem cell graft, following conditioning with treosulfan (30g/m^2^), fludarabine (150mg/m^2^), and alemtuzumab (1mg/kg), and GvHD prophylaxis with ciclosporin A. Four weeks post-infusion, she developed a left hemiparesis; investigations revealed CSF lymphocytosis and DNA polymerase chain reaction was positive for CMV DNA. Magnetic resonance imaging showed evidence of small vessel vasculopathy, consistent with CMV meningoencephalitis. She was treated with foscarnet followed by ganciclovir with neurological recovery and was discharged home. Her post-transplant course was further complicated by the development of autoimmune haemolytic anaemia, requiring treatment with intravenous immunoglobulin, prednisolone, and sirolimus. Twenty-six months post-HSCT, she has discontinued immunosuppression. She has high mixed donor chimerism (CD3 + : 90%, CD19 + : 32%, CD15 + : 25%), has stopped immunoglobulin replacement, and has made good responses to primary vaccination. At latest follow-up, her skin is normal with no features of OS, and she remains on azithromycin prophylaxis only. Neurologically, she displays delayed development of expressive language with evolving spastic diplegia.

Her latest lymphocyte subset analysis is summarised in Table 1. Whole exome sequencing identified a homozygous variant of unknown significance in *JAK3* (NM_0002153) c.1292G > C, p.Arg431Pro in the SH2 domain. This variant has a CADD score of 22.1 and is absent from the gnomAD population database (Supplementary Figure S1). Comparison of VARITY score for the R431P variant against homozygous *JAK3* variants present in gnomAD v4 shows it has the highest predicted pathogenicity for variants in its protein domain (Supplementary Figure S2). The allele balance for this variant was 1.0, excluding somatic reversion mosaicism. No other pathogenic variants in known IEI genes were identified on whole exome sequencing.

### JAK3^R431P^ Mutation is Associated with Diminished T-Cells and Raised B-Cells in Peripheral Blood

**Fig. 1:**
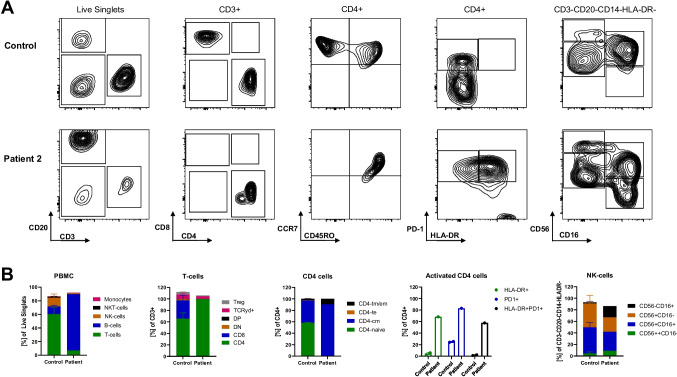
Immunophenotyping of healthy control and patient P2’s peripheral blood cells by flow cytometry. **A** Contour flow plots showing relative abundance of B-cells and reduced T-cells, absence of CD8 + cells and naïve CD4 + cells, hyper activation of CD4 + cells, and skewing of NK-cells towards CD56-CD16 + phenotype in patient P2 with the JAK3^R431P^ variant. **B** Quantification of flow cytometry data in controls and patient P2. Data presented as mean ± SD

Immunophenotyping of two healthy controls and patient P2’s peripheral blood cells by flow cytometry indicated significantly reduced T- and elevated B-cells in P2. Among her T-cells, CD8 + cells and circulating naïve CD4 + cells (CD45RO-CCR7 +) were absent while the frequency of memory CD4 + T-cells was increased. Moreover, we observed a high percentage of circulating activated CD4 + cells in the patient, expressing HLA-DR and/or PD-1. We also noted slightly decreased frequencies of TCRγδ + cells and Treg (CD4 + CD127lowCD25 +). Analysis of NK-cells revealed skewing of CD56lowCD16 + NK-cells toward CD56-CD16 + phenotype, which can be associated with high viral load (Fig. 1A, B) [16–18].

### Preserved Expression of Mutated JAK3^R431P^ Protein

**Fig. 2:**
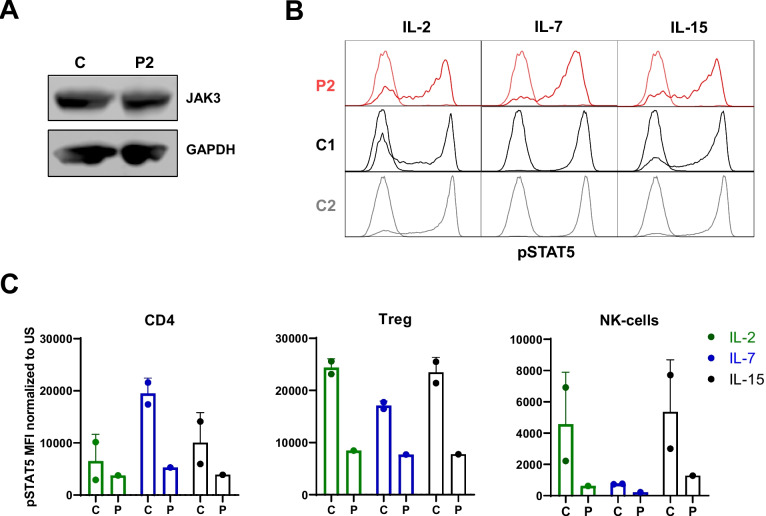
Impaired pSTAT5 response to interleukin IL-2, IL-7, and IL-15 stimulation in patient P2’s PBMC subsets**. A** Unaffected expression of JAK3^R431P^ protein in EBV-LCL cell lines by immunoblotting. **B** Histograms of pSTAT5 in patient P2’s and healthy controls’ (C1, C2) peripheral CD4 cells in response to IL-2, IL-7, and IL-15 stimulation. Dashed area: IL-stimulation, empty line: unstimulated. **C** Impaired pSTAT5 response to interleukin IL-2, IL-7, and IL-15 stimulation in patient P2’s cell subsets. C: Controls C1 and C2, P: patient P2, pSTAT5: phosphorylated STAT5. Data presented as mean ± SD

To further assess the impact of the variant, we measured JAK3 protein expression in P2-derived EBV-LCL cell line by immunoblotting. Expression of the variant JAK3 was equivalent to wild-type JAK3 expressed in EBV-LCL cells derived from healthy control donor (Fig. 2A).

### Hypomorphic Effect of JAK3^R431P^ Variant on its Kinase Activity and JAK-STAT Signalling after Interleukin Stimulation

To further assess any potential hypomorphic effect of this mutation on JAK3 kinase activity, we stimulated PBMC isolated from patient P2 and unaffected individuals with interleukins IL-2, IL-7, and IL-15, and measured their response by downstream STAT5 phosphorylation. The phosphorylation of STAT5 was reduced in patient P2 lymphocytes and NK-cells after stimulation with all three interleukins, IL-2, IL-7, and IL-15, suggesting an impaired kinase activity of normally expressed mutated JAK3^R431P^ protein (Fig. 2B, C; Supplementary Figure S3).

The observation of impaired kinase activity of mutated JAK3 protein was also shown in EBV-LCL cell lines derived from patient P2 and healthy controls. The magnitude of response (MFI) measured by STAT5 phosphorylation after IL-2 and IL-7 stimulation in all EBV-LCL cell lines was too low to draw clear conclusions. However, stimulation with IL-15 suggested a trend towards impaired signalling in patient P2’s EBV-LCL cell line, although in lower magnitude than in data obtained from PBMC (Supplementary Figure S4).

## Discussion

We report two patients with hypomorphic JAK3 deficiency presenting with an OS phenotype and preserved NK-cell production. The *JAK3* variant in P1 (R775H) has been previously reported in two patients. The first report describes a female from Iran with failure to thrive, chronic diarrhoea, and respiratory distress [9]. This patient’s presentation was notable for diffuse erythroderma affecting > 50% of body surface area with acute-on-chronic inflammation extending from the upper to the deep dermis, as well as alopecia. Immunophenotyping demonstrated normal total lymphocyte count, reduced T cell receptor excision circle numbers, and impaired T-lymphocyte proliferation to phytohemagglutinin. However, populations of naïve CD4 + and CD8 + T cells were reportedly normal. Maternal lymphocytes were identified in the patient’s blood at a frequency of 17%, indicative of maternofetal engraftment and thus SCID. The authors demonstrate that the identified R775H variant in *JAK3* is likely to be deleterious, with a low population allele frequency and high predictive scores for pathogenicity using in silico models.

Maternofetal engraftment denotes SCID due to an absence of functional neonatal T cells to identify and reject allogeneic cells. Clinically, maternofetal engraftment and OS are indistinguishable, reflecting their shared pathology of “self”-reactive T cells causing organ damage. Analysis of short tandem repeats from patient lymphocytes is necessary to identify the presence or absence of maternal DNA, and diagnose or exclude maternofetal engraftment. In our patient with the R775H variant, no maternofetal engraftment was identified.

A further case of SCID with this variant was reported by Firtina et al., though it is unclear if this patient had an OS phenotype [15]. The R775H variant is in the pseudokinase domain of *JAK3*. This domain regulates catalytic activity of the kinase domain and therefore modulates its signal transduction activity; mutations in the pseudokinase domain result in normal JAK3 protein expression but a failure to transduce cytokine-dependent signals, possibly due to increased inhibition of kinase activity [19].

Analysis of the novel R431P *JAK3* variant in P2 demonstrated normal expression of the resulting protein. Flow cytometry data indicated that the patient’s JAK3-STAT3 signalling is impaired but not absent in response to IL-2, IL-7, and IL-15 stimulation. This is consistent with the variant’s position in the SH2 domain of JAK3, which is implicated in cytokine receptor binding [20]. Feasibly, differential defects in JAK-STAT signalling downstream of alternative receptors could be contributing to the immunological phenotype, as has been documented for certain hypomorphic *TYK2* variants [21]. For example, IL-15 is not only linked to NK cell development [22], but also CD8 + memory T-cell homeostasis [23]. Its role in maintaining a CD8 + T-cell population has been shown as crucial, potentially contributing to the patient’s CD8 negative status [24–26]. Impaired IL-7 signalling has been linked to atypical JAK3-SCID with preserved NK cell production and impaired T cell function [12, 27].

Defects in signal transduction downstream of IL-7 and IL-15 are understood to lead to the T- and NK- immunophenotype seen in both JAK3- and IL2RG-SCID due to their role in thymic development, differentiation, and expansion of T and NK lymphocytes [12, 22, 25, 28]. Both patients presented with normal total T-lymphocyte counts but extremely elevated CD4:CD8 ratio due to CD8 + lymphopenia, which has normalised following HSCT. Unlike in complete JAK3 deficiency, both patients demonstrated normal NK cell numbers, suggesting the partial kinase activity conferred by their hypomorphic proteins may be sufficient to allow NK cell development.

Rarely, reversion mutations of pathogenic genes back to wild-type may mitigate the deleterious impact of a germline mutation within a somatic clone of that cell line, leading to reversion mosaicism [29]. This may occur through a true “back” mutation, or via a compensatory second-site mutation, and is described in several inborn errors of immunity including SCID caused by pathogenic variants in *ADA*, *IL2RG*, *RAG1*, and *CD3Z*, leading to preserved T-cell numbers and a milder phenotype [29]. Reversion mosaicism has been described in two siblings with hypomorphic JAK3 SCID, one showing reversion mosaicism in both CD4 + and CD8 + T-cells, and one with revertant CD8 + T-cells only [30]. Somatic reversion was excluded in our patient P2 by identification of an allelic balance of 1.0 for the R431P variant.

OS is classically associated with mutations in *RAG1* and *RAG2*, which impair V(D)J recombination and result in expansion of an oligoclonal population of T-cells [31]. However, OS has also been described in other genetic aetiologies that impair lymphocyte development, including *IL7RA, IL2RG, ADA, DCLRE1C, RMRP* and *LIG4* deficiencies*,* and CHARGE syndrome [7, 8, 32–37]. It remains rare and is estimated to represent 5% of cases of SCID, and is typically diagnosed later than classical SCID [38]. The presence of OS was historically associated with a poorer outcome at HSCT, though more recently, a report by the Primary Immune Deficiency Treatment Consortium stratifying SCID patients by ‘typical’ or ‘atypical’ (including OS) features found no impact on survival, rates of GvHD, or immune reconstitution post-HSCT [39, 40]. Historic outcomes for OS patients may relate to pre-HSCT immunosuppression or a tendency for more myeloablative conditioning regimens compared to ‘typical’ SCID: in a previous Primary Immune Deficiency Treatment Consortium study, 46% of patients with ‘atypical’ SCID received myeloablative chemotherapy compared to 19% with ‘typical’ SCID [39]. Today, this added myeloablation typically comes from addition of thiotepa to treosulfan/fludarabine, or use of pharmacokinetically-dosed busulfan [41]. Patients may also require pre-HSCT ciclosporin A or alemtuzumab to reduce organ infiltration by autoreactive T-cells and reduce the risk of GvHD arising from an inflamed milieu at the point of graft infusion.

In conclusion, we report two homozygous hypomorphic JAK3 variants causing SCID with OS and demonstrate impaired signal transduction by the JAK3^R431P^ variant despite normal protein expression. We suggest that JAK3 defects that disrupt but do not abrogate lymphocyte development may lead to OS, similarly to other genetic aetiologies of OS that do not directly impact V(D)J recombination.

### Supplementary Information

Below is the link to the electronic supplementary material.

Supplementary file1 Supplemental Figure S1. Correlation of Combined Annotated Dependent Depletion (CADD) scores with minor allele frequencies (MAF) for the JAK3 variants identified in this report and other homozygous JAK3 variants in gnomAD v4 (PDF 6 KB)

Supplementary file2 Supplemental Figure S2. High predicted pathogenicity for the JAKR431P variant compared to other JAK3 homozygous variants in its protein domain using the VARITY score (PDF 7 KB)

Supplementary file3 Supplemental Figure S3. Impaired pSTAT5 response to interleukin IL-2, IL-7, and IL-15 stimulation in patient P2’s cell subsets. C: Controls C1 and C2, P: patient P2, pSTAT5: phosphorylated STAT5. Data presented as mean ± SD (PDF 66 KB)

Supplementary file4 Supplemental Figure S4. Phospho-STATs signalling after stimulation with IL-2, IL-7, IL-15, and IL-21 in EBV-LCL cell lines showing impaired response of patient P2’s cells. A) Representative flow cytometry histograms of pSTAT5 stimulation in patient P2 and controls (C1, C2). Dashed area: IL-stimulation, empty line: unstimulated. B) Quantification of three independent flow cytometry experiments. Data presented as mean ± SD, ns: non-significant, unpaired t-test (PDF 87 KB)

## Data Availability

The datasets generated during and analysed during the current study are not available for sharing, but the corresponding author may be contacted with queries.

## Abbreviations

BCG: Bacille Calmette-Guérin
C: Control
CMV: Cytomegalovirus
EBV-LCL: Epstein-Barr Virus-bearing lymphoblastoid cell line
FCS: Foetal calf serum
GvHD: Graft-versus-host disease
HHV6: Human herpesvirus-6
Ig: Immunoglobulin
IL: Interleukin
JAK: Janus kinase
MFI: Mean fluorescence intensity
OS: Omenn syndrome
P: Patient
PBMC: Peripheral blood mononuclear cells
RAG: Recombination-activating gene
RPMI medium: Roswell Park Memorial Institute medium
SCID: Severe combined immunodeficiency
TCR: T-cell receptor
