# Supplementary figures and images for: Omenn Syndrome in Two Infants with Different Hypomorphic Variants in Janus Kinase 3
Source: J Clin Immunol. 2024 Apr 10;44(4):98. doi: 10.1007/s10875-024-01699-5 (PMC11006754; doi:10.1007/s10875-024-01699-5)

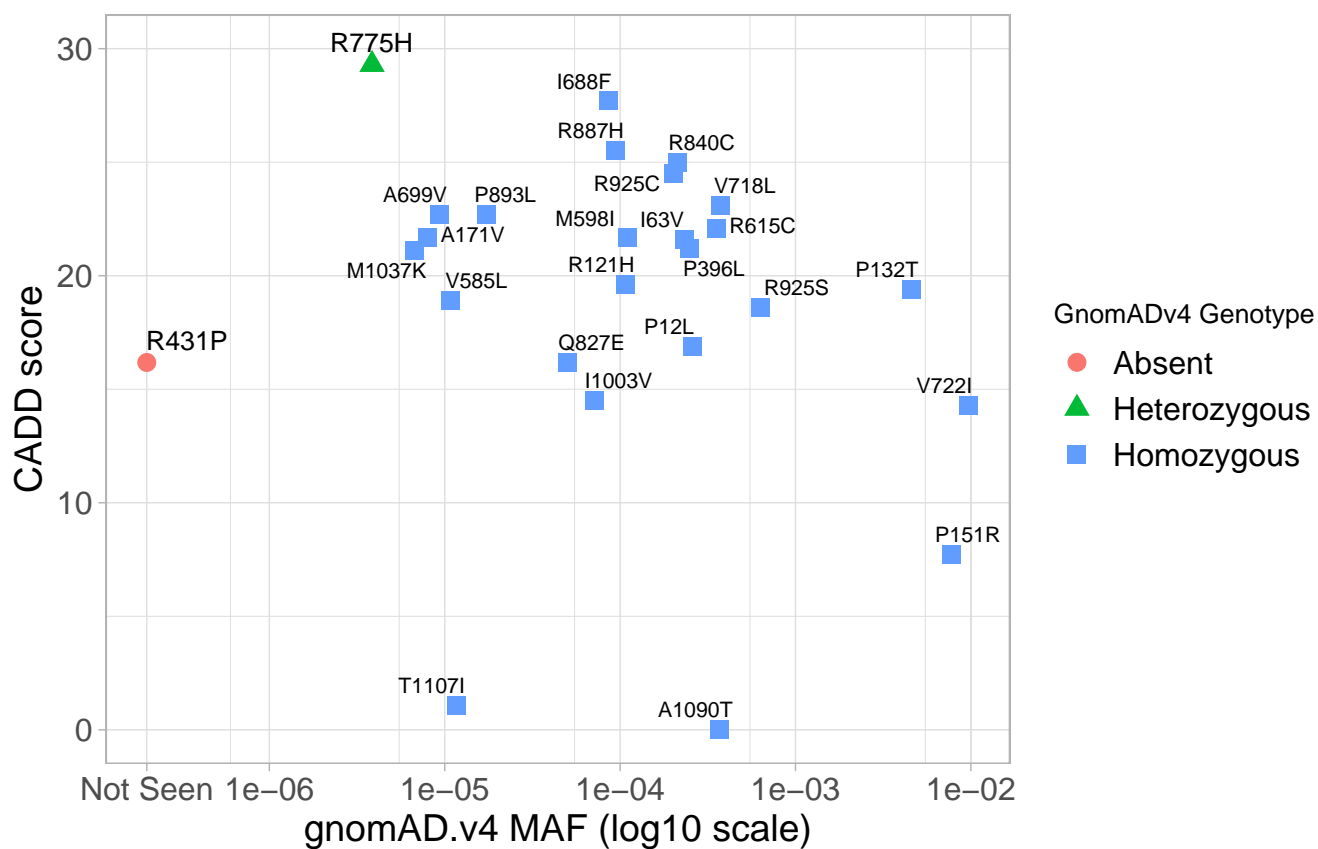

Supplement: Supplementary file 1 — Supplementary file1 Supplemental Figure S1. Correlation of Combined Annotated Dependent Depletion (CADD) scores with minor allele frequencies (MAF) for the JAK3 variants identified in this report and other homozygous JAK3 variants in gnomAD v4 (PDF 6 KB) [file 10875_2024_1699_MOESM1_ESM.pdf]

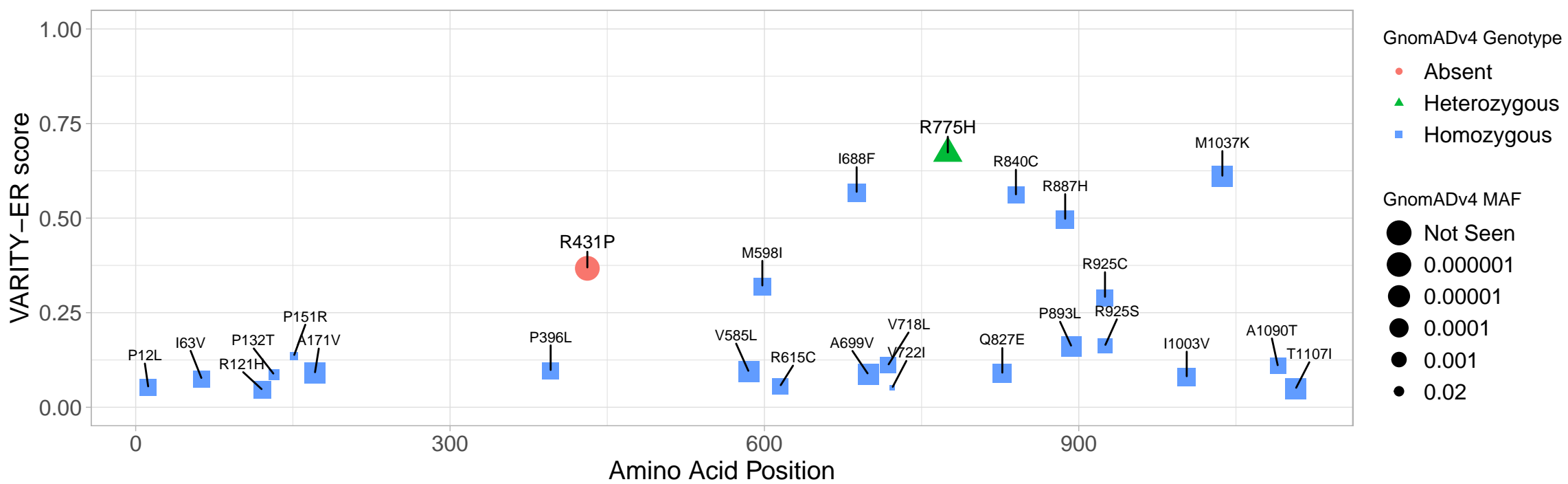

Supplement: Supplementary file 2 — Supplementary file2 Supplemental Figure S2. High predicted pathogenicity for the JAKR431P variant compared to other JAK3 homozygous variants in its protein domain using the VARITY score (PDF 7 KB) [file 10875_2024_1699_MOESM2_ESM.pdf]

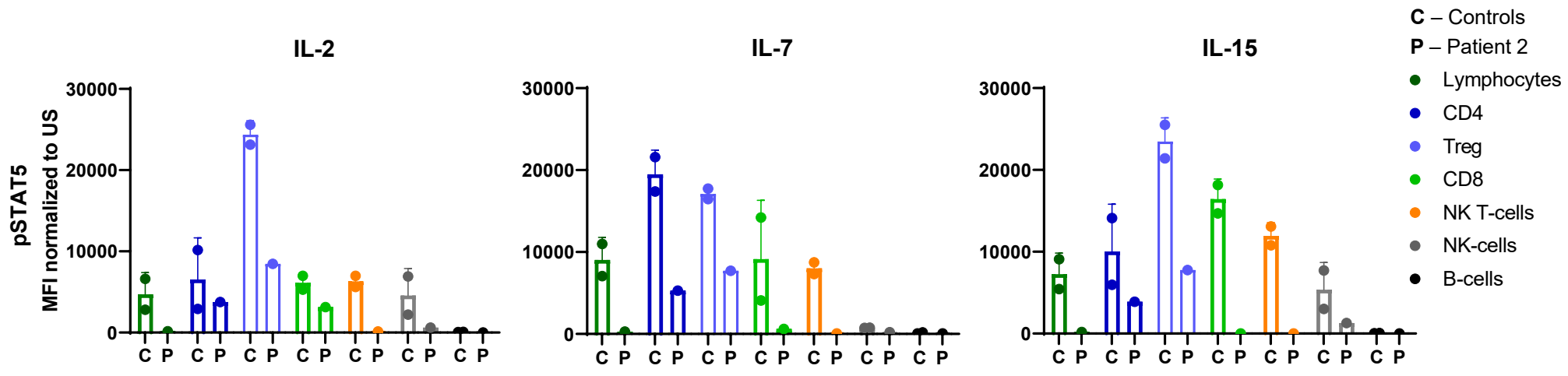

Supplement: Supplementary file 3 — Supplementary file3 Supplemental Figure S3. Impaired pSTAT5 response to interleukin IL-2, IL-7, and IL-15 stimulation in patient P2’s cell subsets. C: Controls C1 and C2, P: patient P2, pSTAT5: phosphorylated STAT5. Data presented as mean ± SD (PDF 66 KB) [file 10875_2024_1699_MOESM3_ESM.pdf]

**A**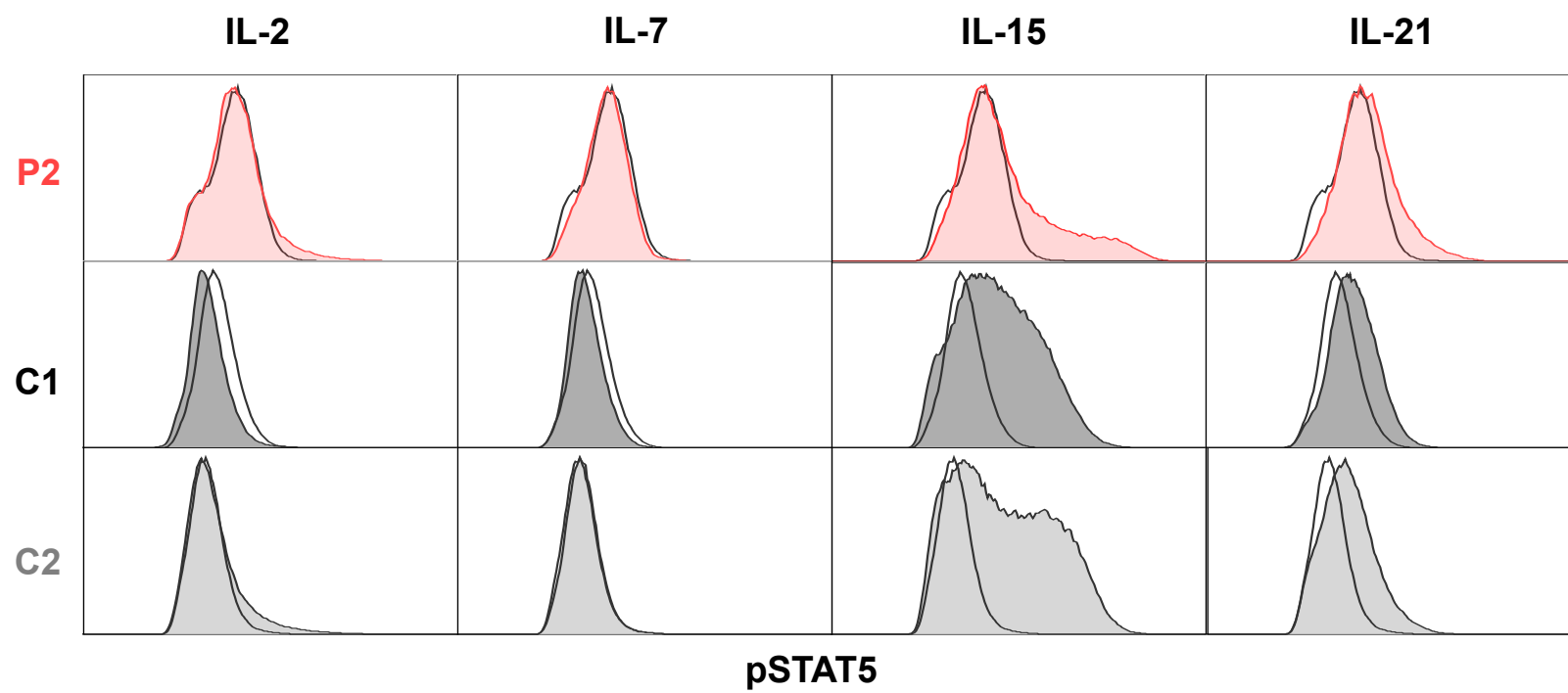**B**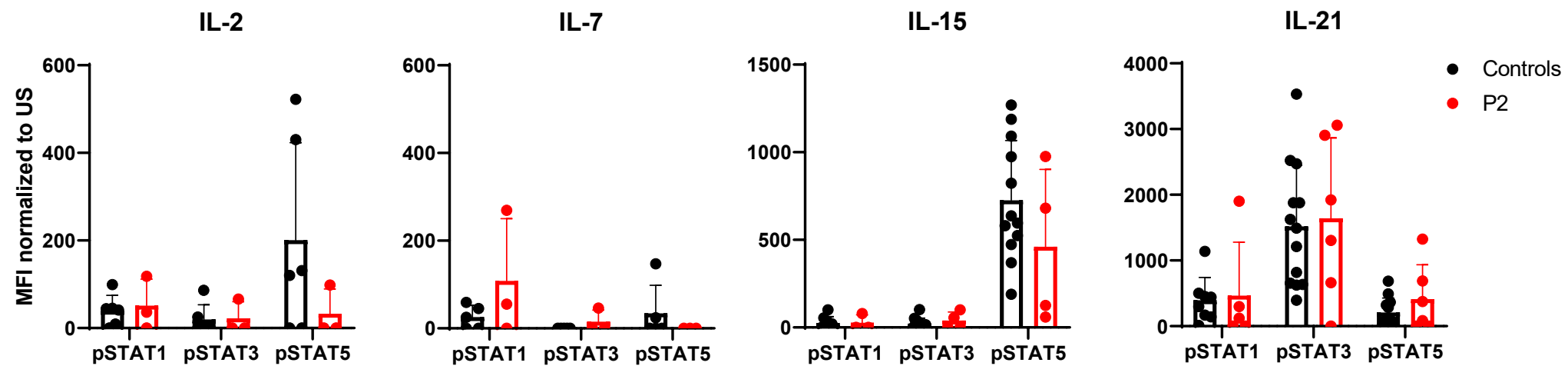

Supplement: Supplementary file 4 — Supplementary file4 Supplemental Figure S4. Phospho-STATs signalling after stimulation with IL-2, IL-7, IL-15, and IL-21 in EBV-LCL cell lines showing impaired response of patient P2’s cells. A) Representative flow cytometry histograms of pSTAT5 stimulation in patient P2 and controls (C1, C2). Dashed area: IL-stimulation, empty line: unstimulated. B) Quantification of three independent flow cytometry experiments. Data presented as mean ± SD, ns: non-significant, unpaired t-test (PDF 87 KB) [file 10875_2024_1699_MOESM4_ESM.pdf]
